# Recombinant Chromosomes Resulting From Parental Pericentric Inversions—Two New Cases and a Review of the Literature

**DOI:** 10.3389/fgene.2019.01165

**Published:** 2019-11-14

**Authors:** Thomas Liehr, Anja Weise, Kristin Mrasek, Monika Ziegler, Niklas Padutsch, Kathleen Wilhelm, Ahmed Al-Rikabi

**Affiliations:** Institute of Human Genetics, Jena University Hospital, Friedrich Schiller University, Jena, Germany

**Keywords:** balanced pericentric inversion, recombinant chromosomes, dosage sensitive genes, duplication, deletion

## Abstract

A balanced pericentric inversion is normally without any clinical consequences for its carrier. However, there is a well-known risk of such inversions to lead to unbalanced offspring. Inversion-loop formation is the mechanism which may lead to duplication or deletion of the entire or parts of the inverted segment in the offspring. However, also partial deletion and duplication may be an effect of a parental inversion, depending on the size of the inversion and the uneven number of crossing over events, also suggested to be due to an inversion loop. Here we describe two new cases of recombinant chromosomes and provide a review of the literature of comparable cases. Interestingly, this survey confirmed the general genetic principle that gain of copy numbers are better tolerated than losses. Furthermore, there is a non-random distribution of all human chromosomes concerning their involvement in recombinant formation, which is also discussed.

## Introduction

As reviewed by Martin (1991) one can find pericentric inversions in human in 1–2% of general population. Normally these balanced chromosomal rearrangements do not cause any problem for the carrier, but during meiosis there is a certain risk of inversion loop formation leading to *de novo* duplication (e.g. Malinverni et al., 2016), deletion (e.g. Lacbawan et al., 1999) or a combination of both in the offspring (**Supplementary Table 1**) when an uneven number of crossing over events occur within the inversion loop. If the latter happens, this is denominated as the formation of a recombinant chromosome. As explained by Morel et al. (2007) for male gametogenesis: "an odd number of crossovers within the loop results in one spermatozoon bearing the normal chromosome, one the inverted chromosome and two recombinants with both duplicated and deficient chromosome segments including the regions distal to the inversion [duplication q/deletion p (dup q/del p) or del q/dup p]". Besides, other rare rearrangements may be due to a parental inversion, like unequal crossing over (Yang et al., 1997), U-loop-formation (Ashley et al., 2007), breakage and unequal reunion of sister chromatids within the inversion loop (Phelan et al., 1993), or even ring-chromosome formation (Hu et al., 2006). Also, recombinants have been seen in triploid fetuses (Ekblom et al., 1993).

**Table 1:** Percentage of maximally deleted and duplicated regions per chromosome arm, compatible with live.

chr.	del/p	dup/p	del/q	dup/q
**X**	82	83	95	83
**1**	n.a.	11	11	n.a.
**2**	4	n.a.	n.a.	10
**3**	10	10	63	63
**4**	90	83	16	58
**5**	75	75	50	50
**6**	24	57	13	31
**7**	8	70	28	94
**8**	65	65	22	80
**9**	35	n.a.	n.a.	29
**10**	87	87	33	98
**11**	n.a.	95	32	n.a.
**12**	15	n.a.	n.a.	10
**13**	n.a.	n.a.	41	85
**14**	n.a.	n.a.	35	35
**15**	n.a.	n.a.	n.a.	32
**16**	36	38	80	80
**17**	20	98	9	15
**18**	88	88	95	95
**19**	27	n.a.	n.a.	28
**20**	23	90	13	43
**21**	n.a.	n.a.	n.a.	73
**22**	n.a.	n.a.	43	57
**average**	**53**	**68**	**40**	**55**

It has been originally suggested that viable, but mentally and physically impaired offspring can only result if the inversion includes less than 30% of the length of the affected chromosome (Martin, 1991). Before, it was proposed to determine the possibility for viable unbalanced offspring by measuring the percentage of haploid autosomal length of the chromosomal segments distal to the inversion-breakpoints (Daniel, 1981). Later on, Morel et al. (2007) suggested that no recombinants can be produced when the inverted segment size is <30%, a few recombinants when inverted segment size is within 30–50% and significant numbers are produced when the inverted segment size is >50% of the total length of the affected chromosome. Nonetheless, also examples were found for recombinants less than 100 Mb in size (Malan et al., 2006) or large families without any recombinant offspring not fitting to that suggested rules (Van der Linden et al., 1975; Honeywell et al., 2012).

Here we report two new cases with pericentric inversion and offspring with recombinant chromosomes and provide a review of overall 210 such cases {plus >100 cases with "recombinant chromosome 8 syndrome" [Online Mendelian Inheritance in Man (OMIM) 179613]}. This data includes also the 56 cases reviewed in 1997 by Ishii and colleagues. As preferential maternal origin of recombinant chromosomes was already shown by Ishii et al. (1997) this was not recapitulated in the present study. Similar effects are well known for small supernumerary marker chromosomes (Liehr 2006) and passing on of other kinds of chromosomal aberrations in human (Liehr et al., 2018). For clinical impact and impact of large and small to submicroscopic paracentric inversions, the latter being part of normal variance in humans, see Pettenati et al. (1995); Liehr et al. (2018) and database of genomic variants (http://dgv.tcag.ca/dgv/app/home).

## Material and Methods

### Cases Studied

The cases include here were identified during routine (molecular) cytogenetic diagnostics and informed consent for publication were provided.

Family 1: Here a healthy female had two affected children with two different male partners. The first son, 17y, showed slight mental impairment, dwarfism, sensorineural hearing loss, and facial dysmorphism; the second son, 6y, also had slight mental impairment, microcephaly, sensorineural hearing loss, dysmorphic signs. Blood samples were available from mother and the two children.

Family 2: Blood and amnion cells of a healthy pregnant female were studied due to sonographic abnormalities detected during routine diagnostics at 16 weeks of gestation.

### Molecular Cytogenetic Tests

Blood and/or amnion from both families were subjected to routine cell culture or DNA-extraction using standard procedures. Metaphase preparation was performed according to standard procedures and karyotypes were analyzed by G-banding at a ∼450 band level. Fluorescence *in situ* hybridization (FISH) was done using probes for subtelomeric regions of chromosome 18 (Abbott, Vysis, Wiesbaden, Germany), partial chromosome paints for the same chromosome (home brewed probes of Liehr and Claussen, 2002) or a multicolor banding probe set for chromosome 11 (Liehr et al., 2002). Array-comparative genomic hybridization (aCGH) was done as previously reported (Coci et al., 2017).

### Literature Search

Born and unborn cases with recombinant chromosomes were put together based on Ishii et al. (1997); Schinzel (2001), and search in https://www.ncbi.nlm.nih.gov/pubmed and https://www.google.de/. *De novo* cases or such with not clarified parental origin were not included here. Definitely cases reported only on genetic meetings were missed, as those are neither provided systematically by any libraries not being available online.

## Results

### Cases Studied

**Figure 1:**
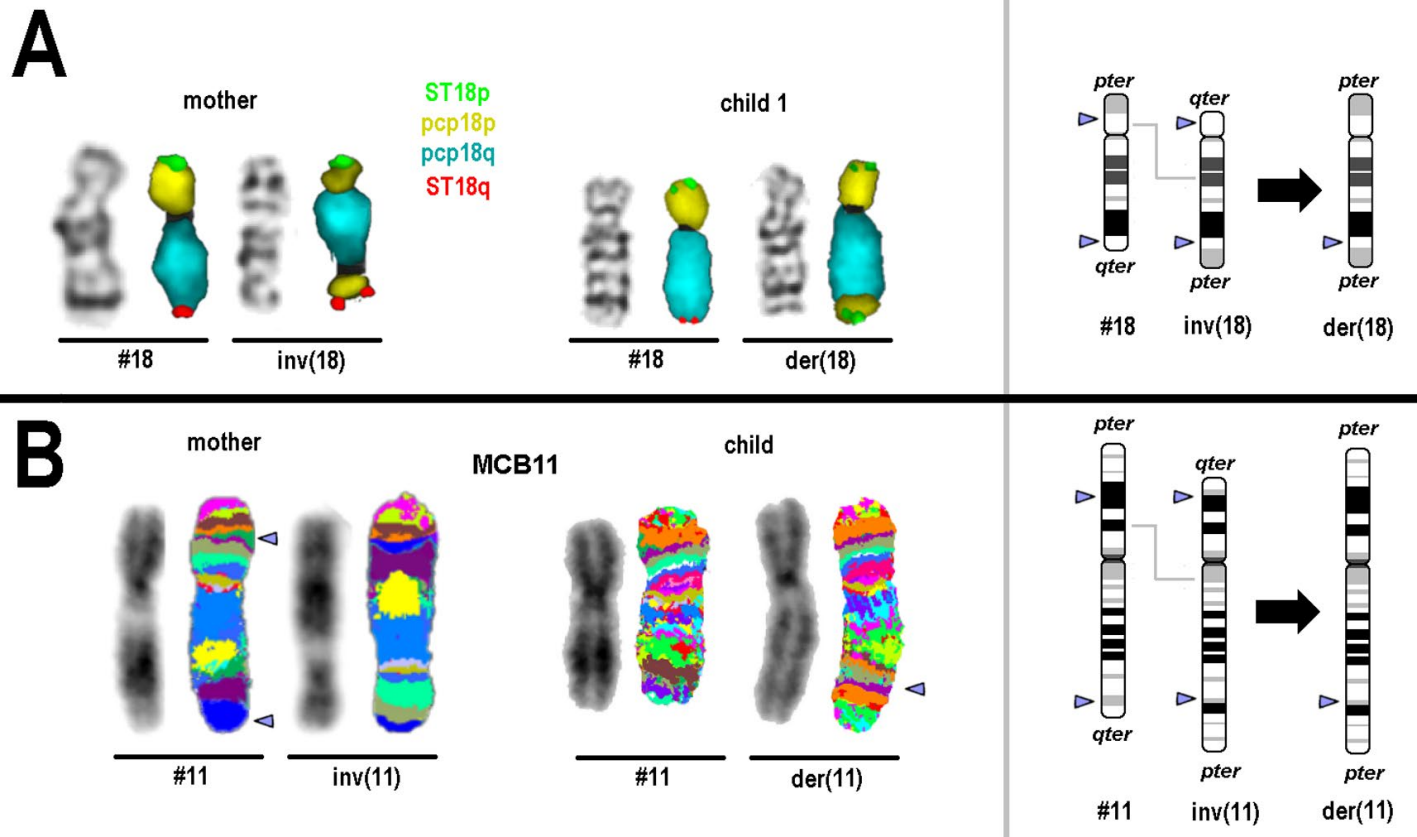
Results of molecular cytogenetics performed for families 1 and 2. On the left side GTG-/inverted 4′,6-diamidino-2-phenylindole-banding result and FISH result of the corresponding normal and derivative chromosome is depicted. On the right schematic depictions of normal, inverted and derivative/recombinant chromosome is shown; breakpoints are highlighted by arrow-heads. **(A)** Normal and derivative chromosomes 18 of mother and child 1 after GTG-banding and FISH are visible. For FISH subtelomeric probes for 18pter (ST 18p) and 18qter (ST18q) and partial chromosome paints (pcps) for 18p and 18q were used. **(B)** Normal and derivative chromosomes 11 of mother and unborn child after inverted 4′,6-diamidino-2-phenylindole-banding and FISH are visible. For FISH a chromosome-11specific multicolor banding probe set was used—results are depicted in two different pseudocolor bandings; the latter had to be applied, due to different preparation qualities of blood and amnion derived chromosomes. Arrowheads highlight the chromosomal breakpoints.

Family 1: The mother was identified to be carrier of a pericentric inversion in chromosome 18; karyotype: 46,XX,inv(18)(p11.22q22.3), while both children had the same recombinant chromosome 18; karyotype: 46,XY,rec(18)(pter->q22.3::p11.22->pter).arr[GRCh37] 18p11.32p11.22(118760_9774819)x3,18q22.3q23(69934975_​78010032)x1 (**Figure 1A**).

**Figure 2:**
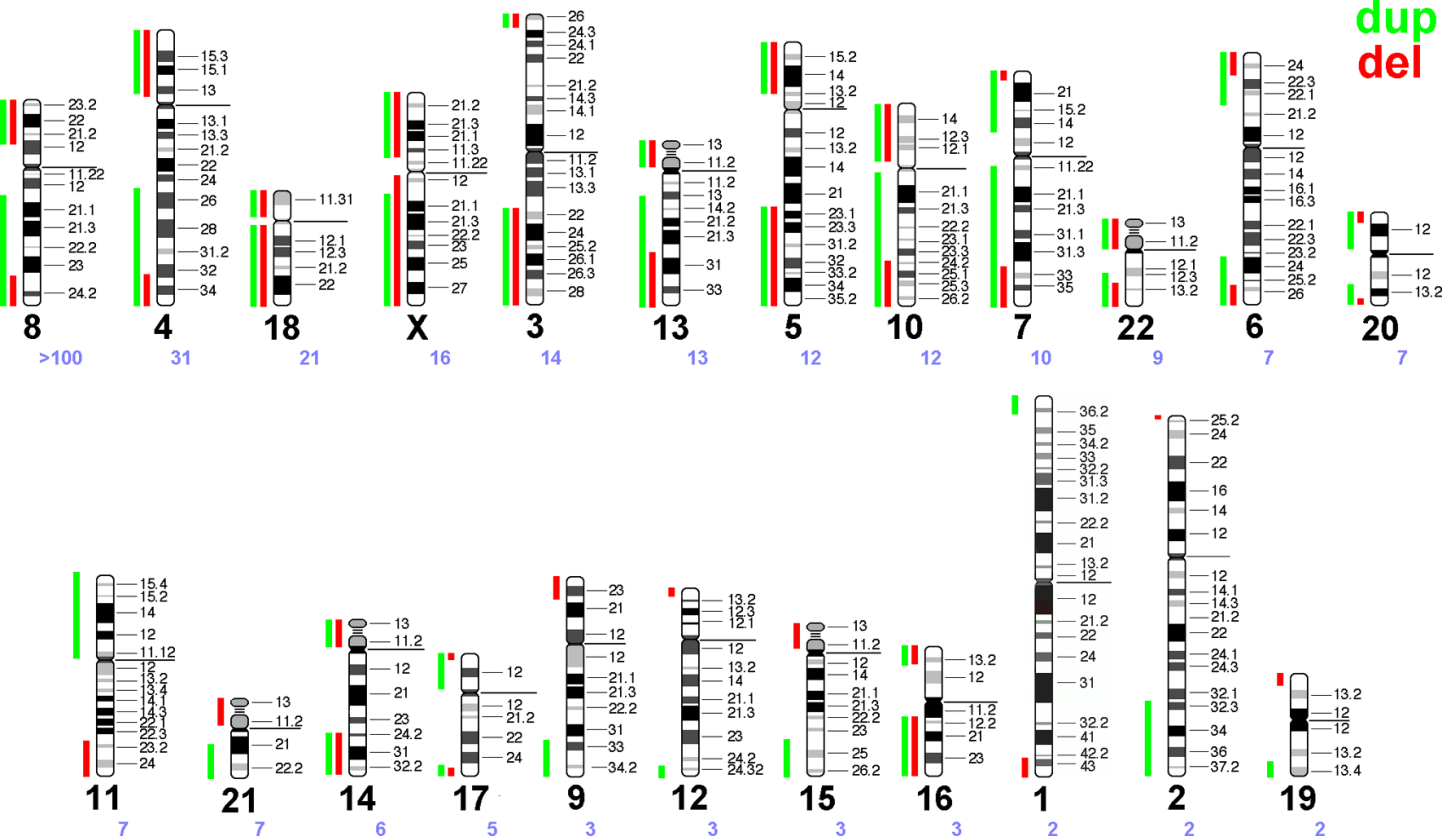
Summary of the literature survey (see **Supplementary Table 1**). Maximal regions of terminal gains or losses along each human autosome and the X-chromosome are entered as green and red vertical lines, each. Chromosomes are sorted according to the number of cases reported with a corresponding recombinant chromosome due to a parental pericentric inversion—the chromosome number is given as a large black and the number of reported cases as a small violet number below each idiogram.

Family 2: in the mother a karyotype 46,XX,inv(11)(p14.3q24) was identified and the unborn child was carrier of a karyotype: 46,XX,rec(11)(pter->q23?.3::p14.3->pter) (**Figure 1B**).

### Literature Search

Literature search revealed overall 210 families/cases with recombinant chromosomes due to an inherited balanced pericentric inversion [**Supplementary Table 1**—plus >100 cases with "recombinant chromosome 8 syndrome" (OMIM 179613)]. Examples for all chromosomes were found, apart from Y-chromosome.

Recombinant chromosomes provide terminal deletions and duplications to the human genome. Based on **Supplementary Table 1** a scheme was drawn in **Figure 2** highlighting the terminal deletions and duplications being compatible with human live.

## Discussion

Two new cases of recombinant chromosomes (rec) were added to the yet reported 210 comparable cases [plus >100 cases with "recombinant chromosome 8 syndrome" (OMIM 179613)], which all are due to a parental pericentric inversion. In contrast to the review of Ishii et al. from 1997 now there are examples available for all human chromosomes, apart for Y-chromosome. However, rec(Y) chromosomes should only be possible in case of men with 2 Y-chromosomes, one with pericentric inversion; and such an instant was not reported yet.

Interestingly, a well-known principle of copy number variants (CNVs) can also be deduced from **Figure 2**: gains of copy numbers are better compensable by human genome than losses; examples are microdeletion/microduplication syndromes or the fact that only trisomies 13, 18, and 21 are viable but not monosomies of those three chromosomes (Weise et al., 2012). In this study (**Figure 2**) for practically all chromosomes the regions compatible with live are lager for gains than for losses of copy numbers (see also **Table 1**).

The observed frequency of recombinants is chromosome-specific, as well as differences concerning arising of viable recombinant chromosomes are different:

– Chromosomes with more than 50 reports summarized in this study:Chromosome 8 is the only one with >100 reported cases and even an own OMIM number for a syndrome caused by this kind of rearrangement. Most likely this is due to a high frequency of inv(8)(p23.1q22.1) in Hispanic population in USA (Sujansky et al., 1993).
– Chromosomes with more than 7-10 reports summarized in this study:Cases involving chromosomes 4 and 5 may have been more frequently observed due to more detailed cytogenetic studies in patients with Wolf-Hirschhorn- (OMIM 194190) and Cri-du-Chat-syndrome (OMIM 123450), respectively. Chromosomes 13, 18, and 21 are the gene-poorest human chromosomes, thus, partial deletions in them are more tolerable than for other autosomes. Chromosomes 7 and 11 underlay imprinting and thus are also connected with well-known imprinting disorders [Wiedemann-Beckwith- (OMIM 130650) and Silver-Russel-syndrome (OMIM 180860)]; thus, patients with these disorders also may be studied more likely in detail than others. X-chromosome aberrations may lead to problems with sex-determination and/or infertility—thus also such aberrations are more likely to be picked up than other (autosomal) ones. For chromosomes 3 and 10, where recombinants are also regularly observed (**Figure 2**) these two chromosomes have in some populations regularly appearing large pericentric inversions [inv(3)(p25q23), inv(3)(p25q25), or inv(10)(p11q26) (Gardner and Amor 2018)], like reported for chromosome 8 in Hispanic population in USA.
– Chromosomes only rarely observed summarized in this study: Among remainder chromosomes some are relatively gene-rich (like chromosomes 1 and 19) or acrocentrics (in which pericentric inversions are quite rare, chrs. 13,14, 15, 21, and 22).

Besides the yet discussed factors potentially influencing the frequencies of recombinant chromosomes in viable human offspring, different recombination rates and recombination hot-spots along each chromosome and in dependence of the gender meiosis is going through as nicely outlined by Bhatt et al. (2014) may also have an impact here.

According to Gardner and Amor (2018) formation of recombinant chromosomes in gametes of pericentric inversion carriers is a function of the size of the inversion: the larger the inversion, the more frequently recombinants are observable in the gametes. Also, p-deletion/q-duplication- appear about in same frequencies as q-deletion/p-duplication-recombinants. However, as already suggestable from data of Ishii et al. (1997), p-deletion/q-duplication is about double as frequent than q-deletion/p-duplication in viable forms of recombinant chromosomes. Considering the before discussed difference of CNVs, being present as gains or as losses, one would have to consider in general lower numbers of dosage sensitive genes in the p-arm of the human chromosomes than in the q-arms.

Overall, here an up to date review of pericentromeric inversion based viable recombinants is provided. Considering also recent new insights into influence of chromosomal rearrangements on interphase architecture (keyword: topologically associated domains = TADs) (Schrank and Gautier, 2019), as well as of overlap of evolutionary conserved breakpoints (important in speciation) and breakpoints observed in clinical cases (Liehr et al., 2011), the importance of gross cytogenetic aberrations to provide a better understanding of general principles of the human genome is highlighted.

## Data Availability Statement

The datasets for this study can be requested from the authors.

## Ethics Statement

Ethical review and approval was not required for the study on human participants in accordance with the local legislation and institutional requirements. The patients/participants provided their written informed consent to participate in this study.

## Author Contributions

TL drafted the paper, and did the literature search. AW did the cytogenetic analyses of family 1. KM did the aCGH analyses of family 1. MZ, NP, and AA-R did the FISH analyses of both families. KW provided family 1 with clinical information.

## Conflict of Interest

The authors declare that the research was conducted in the absence of any commercial or financial relationships that could be construed as a potential conflict of interest.
