# Supplementary material for: Recombinant Chromosomes Resulting From Parental Pericentric Inversions—Two New Cases and a Review of the Literature
Source: Front Genet. 2019 Nov 14;10:1165. doi: 10.3389/fgene.2019.01165 (PMC6868022; doi:10.3389/fgene.2019.01165)
Supplement: Supplementary file 1 [file Table_1.docx]

Supplementary Material

# Supplementary Table

**Supplementary Table 1**

Cases with pericentric inversion leading to viable offspring (postnatal and prenatal cases not separated) with recombinant chromosomes. The largest tolerated imbalance per chromosome arm is highlighted for dup-p/del-q in yellow and for del-p/dup-q in violet.

References are given as numbers and are listed below this Table!

Abbreviations: del = deletion; dup = duplication; del/dup = deletion or duplication; dup/del = duplication or deletion; p = short arm of a chromosome; q = long arm of a chromosome

| **Parental inversion** | **distal p-part** | **distal q-part** | **Reference** |
| --- | --- | --- | --- |
| inv(X)(p11.23q26) | dup | del | 93 |
| inv(X)(p22.3q27) | dup | del | 108 |
| inv(X)(p22.1q28) | dup | del | 1 |
| inv(X)(p22.31q22.3) | dup | del | 92 |
| inv(X)(p21q28) | dup | del | 83 |
| inv(X)(p22q22) | dup | del | 91 |
| inv(X)(p22q24) | dup | del | 90 |
| inv(X)(p22q11) | dup | del | 172 |
| inv(X)(p22.31q27.3) | del | dup | 113 |
| inv(X)(p22q13) | del | dup | 136 |
| inv(X)(p11.2q26) | del | dup | 85 |
| inv(X)(p22.3q26.3) | del | dup | 2 |
| inv(X)(p22.3q27.37) | del | dup | 89 |
| inv(X)(p22q13) | del | dup | 82 |
| inv(X)(p22.2q26) | del | dup | 102 |
| inv(X)(p22.3q26.3) | del | dup | 84 |
|  |  |  |  |
| inv(1)(p36.3q43) | dup | del | 154 |
| inv(1)(p36.3q42.3) | dup | del | 162 |
| n.a. | del | dup |  |
|  |  |  |  |
| n.a. | dup | del |  |
| inv(2)(p25q35) | del | dup | 34 |
| inv(2)(p25.3q33.3) | del | dup | 35 |
|  |  |  |  |
| inv(3)(p25q21) | dup/del | del/dup | 126 (in one patient) |
| inv(3)(p25q21) | dup/del | del/dup | 156 |
| inv(3)(p25q23) | del | dup | 190 |
| inv(3)(p25q23) | del | dup | 191 |
| inv(3)(p25q23) | del | dup | 36 |
| inv(3)(p25q25) | del | dup | 37 |
| inv(3)(p25q25) | del | dup | 38 |
| inv(3)(p25q21) | del | dup | 155 |
| inv(3)(p25q25) | del | dup | 157 |
| inv(3)(p25q25) | del | dup | 158 |
| inv(3)(p26q21) | del | dup | 159 |
| inv(3)(p25q21) | del | dup | 160 |
| inv(3)(p25q21) | del | dup | 161 |
| inv(3)(p25q21) | del | dup | 189 |
|  |  |  |  |
| inv(4)(p1?5q3?5) | dup/del | del/dup | 12 |
| inv(4)(p15.32q35) | dup/del | del/dup | 29 |
| inv(4)(p?q?) | dup | del | 97 |
| inv(4)(p14q35.1) | dup | del | 165 |
| inv(4)(p15.2q32.3) | dup | del | 22 |
| inv(4)(p14q35.1) | dup | del | 96 |
| inv(4)(p15.1q35.1) | dup | del | 32 |
| inv(4)(p15.1q35.1) | dup | del | 33 |
| inv(4)(p15.3q34.2) | dup | del | Decipher Patient ID 269158 |
| inv(4)(p14q35) | dup | del | 199 |
| inv(4)(p14q35) | dup | del | 200 |
| inv(4)(p14q35) | dup | del | 201 |
| inv(4)(p13q35) | dup | del | 202 |
| inv(4)(p13q34) | dup | del | 193 |
| inv(4)(p14q35) | del | dup | 11 |
| inv(4)(p15q35) | del | dup | 12 |
| inv(4)(p16q31.3) | del | dup | 23 |
| inv(4)(p15.2q28.2) | del | dup | 24 |
| inv(4)(p15.2q35) | del | dup | 30 |
| inv(4)(p15.2q35) | del | dup | 25 |
| inv(4)(p16q31.3) | del | dup | 26 |
| inv(4)(p16.2q35.1) | del | dup | 27 |
| inv(4)(p16.3q35.2) | del | dup | 28 |
| inv(4)(p13q28) | del | dup | 31 |
| inv(4)(p15.2q32) | del | dup | 144 |
| inv(4)(p16.3q34.3) | del | dup | 145 |
| inv(4)(p15.3-p16q33) | del | dup | 149 |
| inv(4)(p12q35) | del | dup | 148 |
| inv(4)(p16.3q35.2) | del | dup | 150 |
| inv(4)(p15q31) | del | dup | 192 |
| inv(4)(p15.2q25) | del | dup | 194 |
|  | del | dup |  |
| inv(5)(p15.33q35.3) | dup/del | del/dup | 15 |
| inv(5)(p13q22) | dup/del | del/dup | 110 |
| inv(5)(p14q35) | del | dup | 46 |
| inv(5)(p15.1q35.1) | del | dup | 45 |
| inv(5)(p15.1q35.1) | del | dup | 47 |
| inv(5)(p13q33) | del | dup | 44 |
| inv(5)(p15.1q33.3) | del | dup | 39 |
| inv(5)(p15.31q35.1) | del | dup | 6 |
| inv(5)(p15q32) | del | dup | 40 |
| inv(5)(p13q35) | del | dup | 41 |
| inv(5)(p15.3q35) | del | dup | 43 |
| inv(5)(p15.33q35.3) | del | dup | 186 |
|  |  |  |  |
| inv(6)(p23q27) | dup | del | 49 |
| inv(6)(p23q27) | dup | del | 117 |
| inv(6)(p21q27) | dup | del | 169 |
| inv(6)(p21.2q25.3) | dup | del | 166 |
| inv(6)(p23q25.1) | del | dup | 48 |
| inv(6)(p25q25) | del | dup | 167 |
| inv(6)(p23q23) | del | dup | 168 |
|  |  |  |  |
| inv(7)(p15.1q36) | dup | del | 51 |
| inv(7)(p22q32) | dup | del | 9 |
| inv(7)(p15q36) | dup | del | 50 |
| inv(7)(p15.1q34) | dup | del | 147 |
| inv(7)(p13q36) | dup | del | 170 |
| inv(7)(p22q31.3) | del | dup | 123 |
| inv(7)(p22q11.2) | del | dup | 7 |
| inv(7)(p22q22) | del | dup | 19 |
| inv(7)(p22q32) | del | dup | 195 |
|  |  |  |  |
| inv(8)(p12q24.1) | dup | del | 153 |
| inv(8)(p23q22) | del | dup | 141 |
| inv(8)(p23.3q24.1) | del | dup | 135 |
| inv(8)(p23.1q22.1) | del | dup | 101 |
| inv(8)(p23.1q22.1) | del | dup | 125/ 127 – >100 cases! |
| inv(8)(p22q22) | del | dup | 9 |
| inv(8)(p23q22) | del | dup | 53 |
| inv(8)(p22q24.3) | del | dup | 109 |
| inv(8)(p23q22) | del | dup | 139 |
| inv(8)(p23.3q24.1) | del | dup | 52 |
| inv(8)(p23q22) | del | dup | 54 |
| inv(8)(p23.2q22.3) | del | dup | 95 |
| inv(8)(p23.1q22.1) | del | dup | 122 |
| inv(8)(p23.3q21.11) | del | dup | 143 |
| inv(8)(p12q13) | del | dup | 196 |
|  |  |  |  |
| n.a. | dup | del |  |
| inv(9)(p24.3q34.11) | del | dup | 99 |
| inv(9)(p24q34.1) | del | dup | 124 |
| inv(9)(p22q32) | del | dup | 197 |
|  |  |  |  |
| inv(10)(p15.1q26.12) | dup/del | del/dup | 152 |
| inv(10)(p11.2q25.2) | dup | del | 58 |
| inv(10)(p11.2q26) | dup | del | 59 |
| inv(10)(p11.2q26) | dup | del | 60 |
| inv(10)(p11.2q26.3) | dup | del | 87 |
| inv(10)(p15.1q25.2) | dup | del | 128 |
| inv(10)(p11q26) | dup | del | 184 |
| inv(10)(p12q25) | dup | del | 55 |
| inv(10)(p?q?) | del | dup | 173 |
| inv(10)(p15q24) | del | dup | 57 |
| inv(10)(p11.2q26.3) | del | dup | 146 |
| inv(10)(p11q26) | del | dup | 56 |
|  |  |  |  |
| inv(11)(p11q25) | dup | del | 61 |
| inv(11)(p13q23.3) | dup | del | 62 |
| inv(11)(p15.5q24.3) | dup | del | 118 |
| inv(11)(p15q24) | dup | del | 174 |
| inv(11)(p15.5q25) | dup | del | 175 |
| inv(11)(p15.3q24.1) | dup | del | 204 |
| inv(11)(p14.3q24) | dup | del | Present case 2 |
| n.a. | del | dup |  |
|  |  |  |  |
| n.a. | dup | del |  |
| inv(12)(p13.3q24.31) | del | dup | 142 |
| inv(12)(p13.31q24.31) | del | dup | 182 |
| inv(12)(p13q24.3) | del | dup | 176 |
|  |  |  |  |
| inv(13)(p11q22) | dup/del | del/dup | 137 |
| inv(13)(p11q22) | dup/del | del/dup | 171 |
| inv(13)(p12q22) | dup/del | del/dup | 177 |
| inv(13)(p11q22) | dup | del | 138 |
| inv(13)(p11q21) | del | dup | 115 |
| inv(13)(p13q21) | del | dup | 63 |
| inv(13)(p11q21) | del | dup | 64 |
| inv(13)(p12q13) | del | dup | 65 |
| inv(13)(p11q22) | del | dup | 114 |
| inv(13)(p13q22) | del | dup | 130 |
| inv(13)(p12q14) | del | dup | 66 |
| inv(13)(p11q22) | del | dup | 67 |
| inv(13)(p12q22) | del | dup | 129 |
|  |  |  |  |
| inv(14)(p11.1q24) | dup | del | 149 |
| inv(14)(p11.2q32.1) | del | dup | 107 |
| inv(14)(p12q31) | del | dup | 98 |
| inv(14)(p11.2q24) | del | dup | 3 |
| inv(14)(p11q31) | del | dup | 140 |
| inv(14)(p11q24) | del | dup | 178 |
|  |  |  |  |
| n.a. | dup | del |  |
| inv(15)(p11.2q24) | del | dup | 179 |
| inv(15)(p11.2q25) | del | dup | 180 |
| inv(15)(p12q25.3) | del | dup | 181 |
|  |  |  |  |
| inv(16)(p13.1q22) | dup | del | 68 |
| inv(16)(p13q22) | del | dup | 69 |
| inv(16)(p13q22) | del | dup | 183 |
|  |  |  |  |
| inv(17)(p11q25) | dup | del | 71 |
| inv(17)(p13.3q25.1) | dup | del | 163 |
| inv(17)(p13.3q25.1) | dup | del | 185 |
| inv(17)(p13.3q25.3) | del | dup | 9 |
| inv(17)(p13.3q25.1) | del | dup | 70 |
|  |  |  |  |
| inv(18)(p11.22q23) | dup/del | del/dup | 16 |
| inv(18)(p11.2q22) | dup/del | del/dup | 20 |
| inv(18)(p11q21) | dup/del | del/dup | 132 |
| inv(18)(p11.2q22) | dup/del | del/dup | 198 |
| inv(18)(p12q12) | dup | del | 131 |
| inv(18)(p11.21q22.1) | dup | del | 18 |
| inv(18)(p11q12) | dup | del | 133 |
| inv(18)(p11q21) | dup | del | 134 |
| inv(18)(p11.21q22.3) | dup | del | Present case 1 |
| inv(18)(p11.31q21.33) | dup | del | 151 |
| inv(18)(p11.2q21) | del | dup | 4 |
| inv(18)(p11q11) | del | dup | 72 |
| inv(18)(p11.32q22) | del | dup | 104 |
| inv(18)(p11.2q21.3) | del | dup | 73 |
| inv(18)(p11.2q21.2) | del | dup | 21 |
| inv(18)(p11.2q12.2) | del | dup | 74 |
| inv(18)(p11.2q21.3) | del | dup | 86 |
| inv(18)(p11.21q21.31) | del | dup | 88 |
| inv(18)(p11.31q11.2) | del | dup | 121 |
| inv(18)(p11.2q21.2) | del | dup | 187 |
| inv(18)(p11.2q21.1) | del | dup | 188 |
|  |  |  |  |
| n.a. | dup | del |  |
| inv(19)(p13.3q13.3) | del | dup | 13 |
| inv(19)(p13.3q13.43) | del | dup | 106 |
|  |  |  |  |
| inv(20)(p13q13.3) | dup/del | del/dup | 8 |
| inv(20)(p11.2q13.3) | dup | del | 100 |
| inv(20)(p12q13.3) | dup | del | 75 |
| inv(20)(p13q13.12~13.33) | del | dup | 94 |
| inv(20)(p12.2q13.33) | dup | del | 103 |
| inv(20)(p13q13.1) | del | dup | 112 |
| inv(20)(p13q13.33) | del | dup | 105 |
|  |  |  |  |
| n.a. | dup | del |  |
| inv(21)(p12q21.1) | del | dup | 119 |
| inv(21)(p11q21) | del | dup | 76 |
| inv(21)(p12q22.1) | del | dup | 5 |
| inv(21)(p11.2q22.1) | del | dup | 42 |
| inv(21)(p12q22) | del | dup | 77 |
| inv(21)(p11q22) | del | dup | 120 |
| inv(21)(p11q22) | del | dup | 164 |
|  |  |  |  |
| inv(22)(p11.2q13.31) | dup | del | 10 |
| inv(22)(p11q21) | dup | del | 80 |
| inv(22)(p13q13.1) | del | dup | 14 |
| inv(22)(p13q12) | del | dup | 17 |
| inv(22)(p13q12) | del | dup | 78 |
| inv(22)(p13q12.2) | del | dup | 79 |
| inv(22)(p13q12) | del | dup | 81 |
| inv(22)(p11q13.1) | del | dup | 111 |
| inv(22)(p13q12.2) | del | dup | 116 |
| inv(22)(p13q13.2) | del | dup | 203 |

1. Papoulidis I, Vetro A, Paspaliaris V, Ziegler M, Kreskowski K, Daskalakis G, Papadopoulos V, Dagklis T, Liehr T, Thomaidis L, Manolakos E. A girl with 10 Mb distal Xp deletion arising from maternal pericentric inversion: Clinical data and molecular characterization. Curr Genomics (2018) 19:240-46.
2. Chen CP, Chen CY, Chern SR, Wu PS, Chen YN, Chen SW, Lee CC, Town DD, Lee MS, Yang CW, Wang W. Molecular cytogenetic characterization of Xp22.32→pter deletion and Xq26.3→qter duplication in a male fetus associated with 46,Y,rec(X)dup(Xq) inv(X)(p22.3q26.3), a hypoplastic left heart, short stature, and maternal X chromosome pericentric inversion. Taiwan J Obstet Gynecol (2016) 55:705-11.
3. Kurtulgan HK, Özer L, Yıldırım ME, Ünsal E, Aktuna S, Baltacı V, Akkuş N, Sezgin İ. Recombinant chromosome with partial 14 q trisomy due to maternal pericentric inversion. Mol Cytogenet 2015; 8:92. doi: 10.1186/s13039-015-0195-7
4. Zamani AG, Acar A, Durakbasi-Dursun G, Yildirim MS, Ceylaner S, Tuncez E. Recurrent proximal 18p monosomy and 18q trisomy in a family due to a pericentric inversion. Am J Med Genet A (2014) 164A:1239-44.
5. Oliveira R, Dória S, Madureira C, Lima V, Almeida C, Pinho MJ, Ramalho C, Matoso E, Barros A, Carreira IM, Moura CP. Inv21p12q22del21q22 and intellectual disability. Gene 2013 517:120-4.
6. Ohnuki Y, Torii C, Kosaki R, Yagihashi T, Sago H, Hayashi K, Yasukawa K, Takahashi T, Kosaki K. Cri-du-Chat syndrome cytogenetically cryptic recombination aneusomy of chromosome 5: Implications in recurrence risk estimation. Mol Syndromol (2010) 1:95-8.
7. Tchirikov M, Merinsky A, Strohner M, Bonin M, Beyer V, Haaf T, Bartsch O. Prenatal diagnosis of a recombinant chromosome 7 resulting in trisomy 7q11.22 --> qter. Am J Med Genet A (2010) 152A:721-5.
8. Descipio C, Morrissette JD, Conlin LK, Clark D, Kaur M, Coplan J, Riethman H, Spinner NB, Krantz ID. Two siblings with alternate unbalanced recombinants derived from a large cryptic maternal pericentric inversion of chromosome 20. Am J Med Genet A (2010) 152A:373-82.
9. Grati FR, Chinetti S, Malgara R, Rognoni G, Grimi B, De Toffol S, Milani S, Dulcetti F, Frascoli G, Di Meco AM, Liuti R, Trotta A, Coffa J, Maggi F, Simoni G. Prenatal detection by subtelomeric FISH and MLPA of unbalanced meiotic recombinants resulting from parental pericentric inversions. Mol Cell Probes (2008) 22:316-9.
10. Tagaya M, Mizuno S, Hayakawa M, Yokotsuka T, Shimizu S, Fujimaki H. Recombination of a maternal pericentric inversion results in 22q13 deletion syndrome. Clin Dysmorphol (2008) 17:19-21.
11. Stembalska A, Laczmanska I, Schlade-Bartusiak K, Czemarmazowicz H, Murawski M, Sasiadek M. Recombinant chromosome 4 resulting from a maternal pericentric inversion in two sisters presenting consistent dysmorphic features. Eur J Pediatr (2007) 166:67-71.
12. Garcia-Heras J, Martin J. A rec(4) dup 4p inherited from a maternal inv(4)(p15q35): case report and review. Am J Med Genet (2002) 109:226-30.
13. López-Exposito I, Guillén-Navarro E, Bafallíu JA, Bernabé MC, Escalona A, Fuster C. Duplication 19q13-qter and deletion 19p13-pter arising from an inversion (19)(p13.3q13.3) of maternal origin. Eur J Med Genet (2006) 49:511-5.
14. Boyd LJ, Livingston JS, Brown MG, Lawce HJ, Gilhooly JT, Wildin RS, Linck LM, Magenis RE, Pillers DA. Meiotic exchange event within the stalk region of an inverted chromosome 22 results in a recombinant chromosome with duplication of the distal long arm. Am J Med Genet A (2005) 138:355-60.
15. Bocian E, Suchenek K, Obersztyn E, Nowakowska B, Mazurczak T. Recombination aneusomy of subtelomeric regions of chromosome 5, resulting from a large familial pericentric inversion inv(5)(p15.33q35.3). J Appl Genet (2005) 46:109-14.
16. Vermeulen SJ, Speleman F, Vanransbeeck L, Verspeet J, Menten B, Verschraegen-Spae MR, Wilde PD, Messiaen L, Michaelis RC, Leroy JG. Familial pericentric inversion of chromosome 18: behavioral abnormalities in patients heterozygous for either the dup(18p)/del(18q) or dup(18q)/del(18p) recombinant chromosome. Eur J Hum Genet (2005) 13:52-8.
17. Barajas-Barajas LO, Valdez LL, Gonzalez JR, García-García C, Rivera H, Ramírez L. Sensorineural deafness in two infants: a novel feature in the 22q distal duplication syndrome. Cardinal signs in trisomies 22 subtypes. Genet Couns (2004) 15:167-73.
18. Roberts D, Sweeney E, Walkinshaw S. Congenital cystic adenomatoid malformation of the lung coexisting with recombinant chromosome 18. A case report. Fetal Diagn Ther (2001) 16:65-7.
19. Ishii F, Fujita H, Nagai A, Ogihara T, Kim HS, Okamoto R, Mino M. Case report of rec(7)dup(7q)inv(7)(p22q22) and a review of the recombinants resulting from parental pericentric inversions on any chromosomes. Am J Med Genet (1997) 73:290-5.
20. Mejía-Baltodano G, Bobadilla L, Gonzalez RM, Barros-Núñez P. High recurrence of recombinants in a family with pericentric inversion of chromosome 18. Ann Genet (1997) 40:164-8.
21. Lee MJ, Park SH, Shim SH, Moon MJ, Cha DH. Prenatal diagnosis and molecular cytogenetic characterization of partial dup(18q)/del(18p) due to a paternal pericentric inversion 18 in a fetus with multiple anomalies. Taiwan J Obstet Gynecol (2019) 58:318-23.
22. Wu Y, Wang Y, Wen SW, Zhao X, Hu W, Liu C, Gao L, Zhang Y, Wang S, Yang X, He B, Cheng W. Recombinant chromosome 4 in two fetuses - case report and literature review. Mol Cytogenet (2018) 11:48. doi: 10.1186/s13039-018-0393-1
23. de la Flor Bru J, Guiltart M. Wolf’s syndrome due to pericentric inversion of maternal chromosome 4. An Esp Pediatr (1987) 27:205-7.
24. Villa A, Urioste M, Carrascosa MC, Vázquez S, Martínez A, Martínez-Frías ML. Pericentric inversions of chromosome 4 : report of a new family and review of the literature. Clinc Genet (1995) 48:255-60.
25. Ogle R, Sillence DO, Merrick A, Ell J, Lo B, Robson L, Smith A. The Wolf-Hirschhorn syndrome in adulthood: evaluation of a 24-year-old man with a rec(4) chromosome. Am J Med Genet (1996) 65:124-7.
26. Mun SJ, Cho EH, Chey MJ, Shim GH, Shin BM, Lee RK, Ko JK, Yoo SJ. Recombinant chromosome 4 with partial 4p deletion and 4q duplication inherited from paternal pericentric inversion. Korean J Lab Med (2010) 30:89-91.
27. Dufke A, Eggermann K, Balg S, Stengel-Rutkowski S, Enders H, Kaiser P. A second case of inv(4)pat with both recombinants in the offspring: rec dup(4q) in a girl with Wolf-Hirschhorn syndrome and rec dup(4p). Cytogenet Cell Genet (2000) 91:85-9.
28. Malvestiti F, Benedicenti F, De Toffol S, Chinetti S, Höller A, Grimi B, Fichtel G, Braghetto M, Agrati C, Bonaparte E, Maggi F, Simoni G, Grati FR. Recombinant chromosome 4 from a familial pericentric inversion: prenatal and adulthood wolf-hirschhorn phenotypes. Case Rep Genet (2013) 2013:306098.
29. Hirsch B, Baldinger S. Pericentric inversion of chromosome 4 giving rise to dup(4p) and dup(4q) recombinants within a single kindred. Am J Med Genet (1993) 45:5-8.
30. Narahara K, Himoto Y, Yokoyama Y, Kasai R, Hata A, Kikkawa K, Takahashi Y, Wakita Y, Kimura S, Kimoto H. The critical monosomic segment involved in 4p- syndrome: a high-resolution banding study on five inherited cases. Jinrui Idengaku Zasshi (1984) 29:403-13.
31. Wolf GC, Mao J, Izquierdo L, Joffe G. Paternal pericentric inversion of chromosome 4 as a cause of recurrent pregnancy loss. J Med Genet (1994) 31:153-5.
32. Maurin ML, Labrune P, Brisset S, Le Lorc'h M, Pineau D, Castel C, Romana S, Tachdjian G. Molecular cytogenetic characterization of a 4p15.1-pter duplication and a 4q35.1-qter deletion in a recombinant of chromosome 4 pericentric inversion. Am J Med Genet A (2009) 149A:226-31.
33. Hemmat M, Hemmat O, Anguiano A, Boyar FZ, El Naggar M, Wang JC, Wang BT, Sahoo T, Owen R, Haddadin M. Genotype-phenotype analysis of recombinant chromosome 4 syndrome: an array-CGH study and literature review. Mol Cytogenet (2013) 6:17. doi: 10.1186/1755-8166-6-17.
34. Richter S, Lockwood B, Lockwood D, Allanson J. Abnormal chromosome complement resulting from a familial pericentric inversion. Clin Genet (1989) 39:442-50.
35. Takada F, Matsuura K, Imaizumi K, Kuroki Y. 2q distal trisomy 46,XX,rec(2),dup q,inv(2)(p25.3q33.3)mat. 93th Cong Jpn Pediatr Soc (1990) (Abstr).
36. Sutherland GR, Mulley JC, Goldblatt E. A large kindred with an inv(3)(p25q23): Clinical cytogenetic and genetic marker studies. Ann Genet (1981) 24:202-5.
37. Steinbach P, Adkins WN Jr, Caspar H, Dumars KW, Gebauer J, Gilbert EF, Grimm T, Habedank M, Hansmann I, Herrmann J, Kaveggia EG, Langenbeck U, Meisner LF, Najafzadeh TM, Opitz JM, Palmer CG, Peters HH, Scholz W, Tavares AS, Wiedeking C. The dup(3q) syndrome: Report of eight cases and review of the literature. Am J Med Genet (1981) 10:159-77.
38. Preus M, Vekemans M, Kaplan P. Diagnosis of chromosome 3 duplication q23 -> qter, deletion p25 -> pter in a patient with the C (trigonocephaly) syndrome. Am J Med Genet (1986) 23:935-43.
39. Beemer FA, de France HF, Rosina-Angelista IJM, Gerards LJ, Cats BP, Guyt R. Familial partial monosomy 5p and trisomy 5q: Three cases due to paternal pericentric inversion 5 (p15.1q33.3). Clin Genet (1984) 26:209-15.
40. Schroeder HW Jr, Forbes S, Mack L, Davis S, Norwood TH. Recombination aneusomy of chromosome 5 associated with multiple severe congenital malformations. Clin Genet (1986) 30:285-92.
41. Miyazaki K, Yamanaka T, Asano K. Recombinant(5) with cat cry syndrome due to paternal pericentric inversion. J Pediatr Pract (1986) 30:506-9 (in Jpn).
42. Miyazaki K, Yamanaka T, Ogasawara N. A boy with Down’s syndrome having recombinant chromosome 21 but no SOD-1 excess. Clin Genet (1987) 32:383-7.
43. Kumar D, Heath PR, Blank CE. Clinical manifestations of trisomy 5q. J Med Genet (1987) 24:180-4.
44. Martin AO, Northrup H, Ledbetter DH, Trask B, Van den Engh G, Le Beau MM, Beaudet AL, Gray JW, Sekhon G, Krassikoff N, Booth C. Prenatal detection of 46,XY,rec(5),dup q,inv(5)(p13q33) using DNA analysis, flow cytometry, and in situ hybridization to supplement classical cytogenetic analysis. Am J Med Genet (1988) 31:643-54.
45. Sonoda T, Kawaguchi K, Ohba K, Madokoro H, Ohdo S. Partial monosomy 5p and partial trisomy 5q due to paternal pericentric inversion 5(p15.1q35.1). Jpn J Hum Genet (1989) 34:129-34.
46. Chernos JE, Fowlow SB, Cox DM. Cri du chat syndrome due to meiotic recombination in a pericentric inversion 5 carrier. Clin Genet (1992) 41:266-9.
47. Ono K, Ohashi Y, Nakano H, Togashi H, Kannari Y, Isono S. Partial monosomy 5p and partial trisomy 5q due to paternal pericentric inversion of chromosome 5. Jpn J Hum Genet (1993) 38:319-28.
48. Narahara K, Seno Y, Nishibayashi Y, Hiramoto K, Nanba H, Kikkawa K, Kimoto H. Gene dosage effects for coagulation factors XII (F12) and XIII subunit a (F13A) in a case with partial monosomy 6p resulting from a maternal pericentric inversion of chromosome 6. Jpn J Hum Genet (1987) 32:305-10.
49. Wauters JG, Bossuyt PJ, Roelen L, van Roy B, Dumon J. Application of fluorescence in situ hybridization for early prenatal diagnosis of partial trisomy 6p/monosomy 6q due to a familial pericentric inversion. Clin Genet (1993) 44:262-9.
50. Delicado A, Escribano E, Pajares IL, Diaz de Bustamante A, Carrasco S. A malformed child with a recombinant chromosome 7, rec(7) dup p, derived from a maternal pericentric inversion inv(7)(p15q36). J Med Genet (1991) 28:126-7.
51. Ramer JC, Mowrey PN, Ladda RL. Malformations in a child with dup (7 pter-p15.1) and del (7q36-qter) as a result of familial pericentric inversion. Clin Genet (1991) 39:442-50.
52. Barnes ICS, Kumar D, Bell RJM. A child with a recombinant of chromosome 8 inherited from her carrier mother. J Med Genet (1985) 22:67-70.
53. Smith ACM, Spuhler K, Williams TM, McConnell T, Sujansky E, Robinson A. Genetic risk for recombinant 8 syndrome and the transmission rate of balanced inversion 8 in the Hispanic population of the southwestern United States. Am J Hum Genet (1987) 41:1083-03.
54. Izquierdo LA, McConnell TS, Curet LB, Sarto GE. Recombinant 8 syndrome: The pool of Hispanic pericentric inversion 8 carriers expands numerically and geographically. Am J Obstet Gynecol (1991) 165:1419-22.
55. Yunis E, de Calballero OT. Duplication deficiency as the result of meiotic segregation of a maternal inv(10). Hum Genet (1981) 57:71-4.
56. Lansky-Shafer SC, Daniel WL, Ruiz L. Trisomy 10p produced by recombination involving maternal inversion inv(10)(p11q26). J Med Genet (1981) 18:59-61.
57. Rodriguez MT, Martin MJ, Abrisqueta J. Familial pericentric inversion (10) and its effect on two offspring. J Med Genet (1984) 21:317-9.
58. Ohba K, Ohdo S, Sonoda T. Trisomy 10p syndrome owing to maternal pericentric inversion. J Med Genet (1990) 27:264-6.
59. Kulharya AS, Schneider NR, Wilson GN. Three cases of dup(10p)/del(10q) syndrome resulting from maternal pericentric inversion. Am J Med Genet (1993) 47:817-9.
60. Kozma C, Meck JM. Familial 10p trisomy resulting from a maternal pericentric inversion. Am J Med Genet (1994) 49:281-7.
61. Fryns JP, Haspeslagh M, Goddeeris P, Van Aerde J, Eggermont E, Van den Berghe H. Balanced and unbalanced pericentric inversion of chromosome 11. Ann Genet (1981) 24:182-3.
62. Waziri M, Patil SR, Hanson JW, Bartley JA. Abnormality of chromosome 11 in patients with features of Beckwith-Wiedemann syndrome. J Pediatr (1983) 102:873-6.
63. Wenger SL, Steele MW. Meiotic consequences of pericentric inversions of chromosome 13. Am J Med Genet (1981) 9:275-83.
64. Habedank M. Familial pericentric inversion of chromosome 13 resulting in duplication 13q22->qter. J Med Genet (1982) 19:227-9.
65. Lucas J, Le Mee F, Le Marec B, Pluquailec K, Journel H, Picard F. Trisomy 20p resulting from a maternal pericentric inversion (in French). Ann Genet (1985) 26:187-90.
66. Saadallah N, Hulten M. EM investigations of surface spread synaptonemal complexes in a human male carrier of a pericentric inversion inv(13)(p12q14): The role of heterosynapsis for spermatocyte survival. Ann Hum Genet (1986) 50:369-83.
67. Gordon PL, Dalton JD, Martens PR, Tharapel AT, Wilroy RS. Elucidation of the centromere involvement in an inversion (13) by fluorescent in situ hybridisation. J Med Genet (1993) 30:414-6.
68. Ionasescu V, Patil S, Hart M, Rhead W, Smith W Multiple congenital anomalies syndrome with myopathy in chromosome 16 abnormality. Am J Med Genet (1987) 26:189-94.
69. Bianchi DW, Nicholls RD, Russell KA, Miller WA, Ellin M, Lage JM. Pericentric inversion of chromosome 16 in a large kindred: Spectrum of morbidity and mortality in offspring. Am J Med Genet (1992) 43:791-5.
70. Greenberg F, Stratton RF, Lockhart LH, Elder FFB, Dobyns WB, Ledbetter DH. Familial Miller-Dieker syndrome associated with pericentric inversion of chromosome 17. Am J Med Genet (1986) 23:853-9.
71. Lurie IW, Gurevich DB, Binkert F, Schinzel A. Trisomy 17p11-pter: Unbalanced pericentric inversion, inv(17)(p11q25) in two patients, unbalanced translocations (4;17)(q27;p11) in a newborn and t(4;17)(p15;p11.2) in a fetus. Clin Dysmorphol (1995) 4:25-32.
72. Andrews T, Gardiner AC, Boon AR. Recombinant chromosome 18 in two offspring of a chromosome 18 inversion heterozygote. Ann Genet (1982) 25:185-8.
73. Asano T, Ikeuchi T, Shinohara T, Enokido H, Hashimoto K. Partial 18q trisomy and 18p monosomy resulting from a maternal pericentric inversion, inv(18)(p11.2q21.3). Jpn J Hum Genet (1991) 36:257-65.
74. Ayukawa H, Tsukahara M, Fukuda M, Kondoh O. Recombinant chromosome 18 resulting from a maternal pericentric inversion. Am J Med Genet (1994) 50:323-5.
75. Bown N, Cross I, Davison EV, Burn J. Partial trisomy 20p resulting from a recombination of a familial pericentric inversion. Hum Genet (1986) 74:417-9.
76. Leonard C, Gautier M, Sinet PM, Selva J, Huret JL. Two Down syndrome patients with rec(21),dup q,inv(21)(p11;q2109) from a familial pericentric inversion. Ann Genet (1986) 29:181-3.
77. Fraisse J, Philip T, Bertheas MF, Lauras B. Six cases of partial duplication-deficiency 21 syndrome: 21(dup q22 del p23) due to maternal pericentric inversion inv(21)(p21;q22). A family study. Ann Genet (1986) 29:177-80.
78. Cantu JM, Hernandez A, Vaca G, Plascencia L, Martinez-Basalo C, Ibarra B, Rivera H. Trisomy 22q12 -> qter: ‘‘Aneusomie de recombinaison’’ of a pericentric inversion. Ann Genet (1981) 24:37-40.
79. Fujimoto A, Wilson MG, Towner JW. Duplication of the segment q12.2->qter of chromosome 22 due to paternal inversion 22(p13q12.2). Hum Genet (1983) 63:82-4.
80. Watt JL, Olson IA, Johnston AW, Ross HS, Couzin DA, Stephen GS. A familial pericentric inversion of chromosome 22 with a recombinant subject illustrating a ‘pure’ partial monosomy syndrome. J Med Genet (1985) 22:283-7.
81. Rivera H, Garcia-Esquivel L, Romo MG, Perez-Garcia G, Martinez Y, Martinez R. The 22q distal trisomy syndrome in a recombinant child. Ann Genet (1988) 31:47-9.
82. Buckon KE, Newton MS, Collyer S, Lee M, Spowart G, Scabright M, Sanger R. Phenotypically normal individuals with an inversion(X)(p22q13) and the recombinant (X),dup q. Ann Hum Genet (1981) 45:159-68.
83. Kardon NB, Bronson R, Davis JG, Broekman A, Rosenfeld D. A duplication-deficiency X chromosome resulting from a maternal pericentric inversion. 6th International Congress of Human Genetics, Jerusalem, (1981) p185 (Abstr).
84. Mohandas T, Geller RL, Yen PH, Rosendorff J, Bernstein R, Yoshida A, Shapiro LJ. Cytogenetic and molecular studies on a recombinant human X chromosome: Implications for the spreading of X chromosome inactivation. Proc Natl Acad Sci USA (1987) 84:4954-8.
85. Duckett DP, Young ID. A recombinant X chromosome in a short statured girl resulting from a maternal pericentral inversion. Hum Genet (1988) 79:251-4.
86. Poterico JA, Vásquez F, Chávez-Pastor M, Trubnykova M, Chavesta F, Chirinos J, Salcedo N, Mena R, Cubas S, González R, Alvariño R, Abarca-Barriga H. A Peruvian child with 18p-/18q+ syndrome and persistent microscopic hematuria. J Pediatr Genet (2017) 6:258-66.
87. Chen CP, Chen CY, Chern SR, Wu PS, Chen YN, Chen SW, Lee CC, Town DD, Lee MS, Yang CW, Wang W. Molecular cytogenetic characterization of Xp22.32→pter deletion and Xq26.3→qter duplication in a male fetus associated with 46,Y,rec(X)dup(Xq) inv(X)(p22.3q26.3), a hypoplastic left heart, short stature, and maternal X chromosome pericentric inversion. Taiwan J Obstet Gynecol (2016) 55:705-11.
88. Lustosa-Mendes E, Dos Santos AP, Viguetti-Campos NL, Vieira TP, Gil-da-Silva-Lopes VL. A boy with partial dup(18q)/del(18p) due to a maternal pericentric inversion: Genotype-phenotype correlation and risk of recombinant chromosomes based on systematic review of the literature. Am J Med Genet A (2017) 173:143-50.
89. Kim MK, Seok HH, Kim YS, Chin MU, Sung SR, Lee WS, Shim SH, Yoon TK. Molecular genetic and cytogenetic characterization of a partial Xp duplication and Xq deletion in a patient with premature ovarian failure. Gene (2014) 534:54-9.
90. Nikolis J, Stolević E. Recombinant chromosome as a result of pericentric inversion of X chromosome. Hum Genet (1978) 45:115-22.
91. Madariaga ML, Rivera H. Familial inv(X) (p22q22): ovarian dysgenesis in two sisters with del Xq and fertility in one male carrier. Clin Genet (1997) 52:180-3.
92. Jaillard S, Loget P, Lucas J, Dubourg C, Le Bouar G, Demurger F, Bertorello I, David V, Poulain P, Odent S, Belaud-Rotureau MA. Terminal 6.9 Mb deletion of chromosome 15q, associated with a structurally abnormal X chromosome in a patient with congenital diaphragmatic hernia and heart defect. Eur J Med Genet (2011) 54:186-8.
93. Santiago F, Vieira R, Cordeiro M, Carreira I, Figueiredo A. Late-onset hyperpig-mentation: a case with multi-systemic involvement and recombinant X chromosome. J Eur Acad Dermatol Venereol(2010) 24:84-5.
94. Starr LJ, Truemper EJ, Pickering DL, Sanger WG, Olney AH. Duplication of 20qter and deletion of 20pter due to paternal pericentric inversion: patient report and review of 20qter duplications. Am J Med Genet A (2014) 164A:2020-4.
95. Vera-Carbonell A, López-González V, Bafalliu JA, Piñero-Fernández J, Susmozas J, Sorli M, López-Pérez R, Fernández A, Guillén-Navarro E, López-Expósito I. Pre- and postnatal findings in a patient with a novel rec(8)dup(8q)inv(8)(p23.2q22.3) associated with San Luis Valley syndrome. Am J Med Genet A (2013) 161A:2369-75.
96. Battaglia A, Brothman AR, Carey JC. Recombinant 4 syndrome due to an unbalanced pericentric inversion of chromosome 4. Am J Med Genet (2002) 106:103–6.
97. Wilson MG, Towner JW, Coffin GS, Forsman I. Inherited pericentric inversion of chromosome no. 4. Am J Hum Genet (1970) 22:679-90.
98. Sgardioli IC, Simioni M, Viguetti-Campos NL, Prota JR, Gil-da-Silva-Lopes VL. A new case of partial 14q31.3-qter trisomy due to maternal pericentric inversion. Gene (2013) 523:192-4.
99. Mundhofir FE, Smeets D, Nillesen W, Winarni TI, Yntema HG, de Leeuw N, Hamel BC, Faradz SM, van Bon BW. Monosomy 9pter and trisomy 9q34.11qter in two sisters due to a maternal pericentric inversion. Gene (2012) 511:451-4.
100. Kang JE, Park MY, Cheon CK, Lee HD, Hwang SH, Yi J. A case of partial trisomy 20p resulting from meiotic recombination of a maternal pericentric inversion. Ann Lab Med (2012) 32:91-4.
101. Pickler L, Wilson R, Tsai AC. Revisiting recombinant 8 syndrome. Am J Med Genet A (2011) 155A:1923-9.
102. Breman AM, Ramocki MB, Kang SH, Williams M, Freedenberg D, Patel A, Bader PI, Cheung SW. MECP2 duplications in six patients with complex sex chromosome rearrangements. Eur J Hum Genet (2011) 19:409-15.
103. Wang BT, Hemmat M, Jayakar P, Boyar F, Chan P, El Naggar M, Anguiano A. Paternal mosaic inv(20) resulting in a recombinant chromosome 20 in two siblings. Pediatr Int (2010) 52:492-5.
104. Prontera P, Buldrini B, Aiello V, Rogaia D, Mencarelli A, Gruppioni R, Bonfatti A, Beltrami N, Donti E, Sensi A. Familial pericentric inversion of chromosome 18: intrafamilial variability of the recombinant dup(18q). Genet Couns ( 2010) 21:91-7.
105. Stevens SJ, Smeets EE, Blom E, van Uum CM, Albrechts JC, Herbergs J, Janssen JW, Engelen JJ. Identical cryptic partial monosomy 20pter and trisomy 20qter in three adult siblings due to a large maternal pericentric inversion: detection by MLPA and breakpoint mapping by SNP array analysis. Am J Med Genet A (2009) 149A:2226-30.
106. Schluth-Bolard C, Till M, Rafat A, Labalme A, Le Lorc'h M, Banquart E, Angei C, Cordier MP, Romana SP, Edery P, Sanlaville D. Monosomy 19pter and trisomy 19q13-qter in two siblings arising from a maternal pericentric inversion: clinical data and molecular characterization. Eur J Med Genet (2008) 51:622-30.
107. Sliuzas V, Utkus A, Kucinskas V. Recombinant chromosome 14 due to maternal pericentric inversion. J Appl Genet (2008) 49:205-7.
108. Bleyl SB, Byrne JL, South ST, Dries DC, Stevenson DA, Rope AF, Vianna-Morgante AM, Schoenwolf GC, Kivlin JD, Brothman A, Carey JC. Brachymesomelic dysplasia with Peters anomaly of the eye results from disruptions of the X chromosome near the SHOX and SOX3 genes. Am J Med Genet A (2007) 143A:2785-95.
109. Tokutomi T, Hayashi S, Imai K, Chida A, Ishiwata T, Asano Y, Inazawa J, Nonoyama S. dup(8p)/del(8q) recombinant chromosome in a girl with hepatic focal nodular hyperplasia. Am J Med Genet A (2007) 143A:1334-7.
110. Bartsch O, Ergun MA, Balci S, Kan D, Eggermann T, Kotzot D. Two complementary recombinant chromosomes 5 in a healthy woman. Cytogenet Genome Res (2006) 114:178-82.
111. Hou JW. Trisomy chromosome (22)(q13.1-qter) as a result of paternal inversion (22)(p11q13.1) proved using region-specific FISH probes. Chang Gung Med J (2005) 28:657-61.
112. Grange DK, Garcia-Heras J, Kilani RA, Lamp S. Trisomy 20q13 --> 20qter in a girl with multiple congenital malformations and a recombinant chromosome 20 inherited from a paternal inversion (20)(p13q13.1): clinical report and review of the trisomy 20q phenotype. Am J Med Genet A (2005) 137A:308-12.
113. Kokalj-Vokac N, Marcun-Varda N, Zagorac A, Erjavec-Skerget A, Zagradisnik B, Todorovic M, Gregoric A. Subterminal deletion/duplication event in an affected male due to maternal X chromosome pericentric inversion. Eur J Pediatr (2004) 163:658-63.
114. Phadke SR, Patil SJ. Partial trisomy 13 with features similar to C syndrome. Indian Pediatr (2004) 41:614-7.
115. Bourthoumieu S, Esclaire F, Terro F, Fiorenza M, Eyraud JL, Servaud M, Cantaloube M, Fermeaux V, Yardin C. Two unusual chromosome aberrations ascertained by sonographic anomalies. Prenat Diagn (2004) 24:219-23.
116. Tonk VS, Jesurun CA, Morgan DL, Lockhart LH, Velagaleti GV. Molecular cytogenetic characterization of a recombinant chromosome rec(22)dup(22q)inv(22)(p13q12.2). Am J Med Genet A (2004) 124A:92-5.
117. Anderlid BM, Schoumans J, Annerén G, Sahlén S, Kyllerman M, Vujic M, Hagberg B, Blennow E, Nordenskjöld M. Subtelomeric rearrangements detected in patients with idiopathic mental retardation. Am J Med Genet (2002) 107:275-84.
118. Clarkson B, Pavenski K, Dupuis L, Kennedy S, Meyn S, Nezarati MM, Nie G, Weksberg R, Withers S, Quercia N, Teebi AS, Teshima I. Detecting rearrangements in children using subtelomeric FISH and SKY. Am J Med Genet (2002) 107:267-74.
119. Lazzaro SJ, Speevak MD, Farrell SA. Recombinant Down syndrome: a case report and literature review. Clin Genet (2001) 59:128-30.
120. Tardy EP, Toth A & Kosztolanyi G. Prenatal exclusion of segmental trisomy in familial chromosome 21 pericentric inversion by fluorescence in situ hybridization. Prenat Diag (1997) 17:871-3.
121. Leonard NJ, Tomkins DJ, Demianczuk N. Prenatal diagnosis of holoprosencephaly (HPE) in a fetus with a recombinant (18)dup(18q)inv(18)(p11.31q11.2)mat. Prenat Diagn (2000) 20:947-9.
122. Graw SL, Sample T, Bleskan J, Sujansky E, Patterson D. Cloning, sequencing, and analysis of inv8 chromosome breakpoints associated with recombinant 8 syndrome. Am J Hum Genet (2000) 66:1138-44.
123. Goodman BK, Stone K, Coddett JM, Cargile CB, Gurewitsch ED, Blakemore KJ, Stetten G. Molecular cytogenetic analysis and clinical findings in a newborn with prenatally diagnosed rec(7)dup(7q)inv(7)(p22q31.3)pat. Prenat Diagn (1999) 19:1150-6.
124. Shapira SK, Orr-Urtreger A, Gagos S, Shaffer LG. Constitutional mosaicism for a chromosome 9 inversion resulting in recombinant aneusomy in an offspring. Am J Med Genet (1997) 69:360-4.
125. Sujansky E, Smith AC, Prescott KE, Freehauf CL, Clericuzio C, Robinson A. Natural history of the recombinant (8) syndrome. Am J Med Genet (1993) 47:512-25.
126. Allderdice PW, Ali M, McAlpine PJ. Complementation by two non-homologous recombinant chromosomes 3. Am J Med Genet (1991) 39:396-8.
127. Mattina T, Conti L, Milone G, Marino S, Sorge G. Inv(8)(p23q22) and recombinant derivative in a Sicilian family. Clin Genet (1989) 36:256-61.
128. Roberts P, Williams J, Sills MA. A case of two inversion (10) recombinants in a family. J Med Genet (1989) 26:461-4.
129. Pai GS, Shields SM, Houser PM. Segregation of inverted chromosome 13 in families ascertained through liveborn recombinant offspring. Am J Med Genet (1987) 27:127-33.
130. Maltby EL. Familial pericentric inversion (13) detected by antenatal diagnosis. J Med Genet 1984) 21:149-51.
131. Martin AO, Simpson JL, Deddish RB, Elias S. Clinical implications of chromosomal inversions. A pericentric inversion in No. 18 segregating in a family ascertained through an abnormal proband. Am J Perinatol (1983) 1:81-8.
132. Vigi V, Maraschio P, Basi G, Guerini P, Fracccaro M. Risk for recombinants in pericentric inversions of the (p11q21)region of chromosome 18. Hum Genet (1977) 37:1-5.
133. Teyssier JR, Bajolle F. Duplication-deficiency of chromosome 18, resulting from recombination of a paternal pericentric invesion, with a note for genetic counselling. Hum Genet (1980) 53:195-200.
134. Vianna-Morgante AM, Nozaki MJ, Ortega CC, Coates V, Yamamura Y. Partial monosomy and partial trisomy 18 in two offspring of carrier of pericentric inversion of chromosome 18. J Med Genet (1976) 13:366-70.
135. Lovell M, Herrera J, Coco R. A child with recombinant of chromosome 8 inherited from a carrier mother with a pericentric inversion. Medicina (B Aires) (1982) 42:359-62.
136. Buckton KE, Newton MS, Collyer S, Lee M, Spowart G, Seabright M, Sanger R. Phenotypically normal individuals with an inversion (X) (p22q13) and the recombinant (X), dup q. Ann Hum Genet (1981) 45:159-68.
137. Williamson EM, Miller JF, Seabright M. Pericentric inversion (13) with two different recombinants in the same family.J Med Genet (1980) 17:309-12.
138. Seabright M, Gregson N, Aronson MM, Greene AE, Coriell LL. Recombinant chromosome 13 with a duplication of a short arm. Repository identification No. GM-1570. Cytogenet Cell Genet (1979) 23:281.
139. Fujimoto A, Towner JW, Turkel SB, Wilson MG. A fetus with recombinant of chromosome 8 inherited from her carrier father. Hum Genet (1978) 40:241-8.
140. Trunca C, Opitz JM. Pericentric inversion of chromosome 14 and the risk of partial duplication of 14q (14q31 leads to 14qter). Am J Med Genet (1977) 1:217-28.
141. Fujimoto A, Wilson MG, Towner JW. Familial inversion of chromosome No. 8: an affected child and a carrier fetus. Humangenetik (1975) 27:67-73.
142. Speleman F, Van Roy N, De Vos E, Hilliker C, Suijkerbuijk RF, Leroy JG. Molecular cytogenetic analysis of a familial pericentric inversion of chromosome 12. Clin Genet (1993) 44:156-63.
143. Habhab W, Singer S, Rieß A, Kagan KO, Liehr T, Schäferhoff K, Dufke A, Mau-Holzmann U, Kehrer M. Pre and postnatal findings in a patient with a rec(8)(qter >q21.11 p23.3 >qter) due to a paternal inv(8)(p23.3q21.11). Mol Cytogenet (2019) 12(Suppl 1): 30. P12
144. Polityko A, Shorokh N, Rumyantseva N, Naumchik I, Mrasek K, Liehr T. Rare pericentric inversion of chromosome 4 in parent and rec(4) in child with Wolf-Hirschhorn syndrome: diagnostics by multicolor banding FISH. Abstracts of the 19th Meeting of German Society of Human Genetics (2008): 82, P079.
145. Omori Sarabi S, Behrend C, Mossalaeie MM, Mohseni Moghadam SB, Soleymani M, Kakadezfouli S, Moeini Z, Karimzad Hagh J. A recombinant abnormal gamete resulting from a balanced pericentric inversion of chromosome 4: an affected boy with Wolf-Hirschhorn phenotypes. Cell Journal (2018) 20 (Suppl.1):121 P-153.
146. Dokhanchi A, Bagherizade I, Hadipour F, Vahedi R, Be-hjati F. Report of an abnormal chromosome 10 in a patient with dysmorphism and congenital heart defect (CHD). Cell Journal (2018) 20 (Suppl.1):82 P-163.
147. Sanlaville D, Genevieve D, de Blois M, Faivre L, Morichon N, Amiel J, Gosset P, Picq M, Le Lorch M, Mathieu M, Prieur M, Munnich A, Romana S, Turleau C, Vekemans M. Currarino syndrome and holoproencephaly associated with an inv(7) recombinant chromosome. Europ J Hum Genet (2003) 11(Suppl 1):128; P309.
148. Jemaa B, Chaabouni M, Kessentini M, Meddeb R, Euchi I, Maazoul F, Mrad R, Gaigi S, Chaabouni H. Familiar inversion of chromosome 4 in a Tunisian family resulting in trisomy 4p. Chromosome Research (2009) 17(Suppl 1):S29–S243, 13.77-P.
149. Morales C, Soler A, Bruguera J, Mademont I, Margarit E, Sánchez A. Unexpected prenatal detection of recombinant chromosomes derived from pericentric inversions: report of two cases. Europ J Hum Genet (2008) 16(Suppl 2):P02.155.
150. Malvestiti F, Benedicenti F, De Toffol S, Chinetti S, Höller A, Grimi B, Fichtel G, Braghetto M, Agrati C, Bonaparte E, Maggi F, Simoni G, Grati FR. Recombinant chromosome 4 from a familial pericentric inversion: Prenatal and adulthood Wolf-Hirschhorn phenotypes. Case Rep in Genet (2013) 2013:306098.
151. Kariminejad A, Kariminejad R, Moshtagh A, Zanganeh M, Kariminejad MH, Neuenschwander S, Okoniewski M, Wey E, Schinzel A, Baumer A. Pericentric inversion of chromosome 18 in parents leading to a phenotypically normal child with segmental uniparental disomy 18. Eur J Hum Genet (2011) 19:555-60.
152. Ciuladaite Z, Preiksaitiene E, Utkus A, Kučinskas V. Relatives with opposite chromosome constitutions, rec(10)dup(10p)inv(10)(p15.1q26.12) and rec(10)dup(10q)inv(10)(p15.1q26.12), due to a familial pericentric inversion.Cytogenet Genome Res (2014) 144:109-13.
153. Caer E, Perrin A, Douet-Guilbert N, Amice V, De Braekeleer M, Morel F. Differing mechanisms of meiotic segregation in spermatozoa from three carriers of a pericentric inversion of chromosome 8. Fertil Steril (2008) 89:1637-40.
154. Tuncez E, Balasar Ö, Aydın H. A malformed child with a recombinant chromosome 1 resulting from a maternal large pericentric inversion inv(1)(p36.3q43). Erciyes Med J (2017). 39:S74 (PP-26)
155. Boue J, Hirschhorn K, Lucas M, Gautier M, Moszer M, Bach C. Aneusomies de recombinaision. Conse´quence d’une inversion pericentrique d’un chromosome 3 paternel. Ann Pediatr (1974) 21:567-73.
156. Allderdice PW, Browne N, Murphy DP. Chromosome 3 duplication q21 leads to qter deletion p25 leads to pter syndrome in children of carriers of a pericentric inversion inv(3)(p25q21). Am J Hum Genet (1975) 27:699-718.
157. Fineman RM, Hecht F, Ablow RC, Howard RO, Breg WR. Chromosome 3 duplication q/deletion p syndrome. Pediatrics (1978) 61:611-8.
158. Pope IS, Thuline HC, Aronson MM, Bozarth B, Greene AE, Coriell LL. Duplication of a segment of chromosome 3 in a subject with multiple congenital anomalies and a 47,XYY father, inversion of chromosomes 3 and 9 in the mother, and inversion of chromosome 9 in a brother. Repository identification Nos. GM-1253, GM-1252, and GM-1251. Cytogenet Cell Genet (1979) 24:127-8.
159. Prabhakara K, Bruno DL, Padman P, Prasad S, Sudheer Kumar R, Slater HR, Radha Ramadevi A. Prenatal detection of deletion-duplication of chromosome 3 arising from meiotic recombination of a familial pericentric inversion. Prenat Diagn (2008) 28:466-8.
160. Kawashima H, Maruyama S. A case of chromosome duplicationq deletion syndrome born to mother with a pericentric inversion,inv(3)(p25q21). Jnrui Idengaku Zasshi (1979) 24:9-12
161. Migliori V, Ferrari F, Giamagli CA, Di Stefano P, Galanti E,Guastaferro N. A case of 3q21-qter trisomy and 3p25-termonosomy syndrome. Pediatr Med Chir (1983) 5:237-9.
162. Wettwer M. Molekularzytogenetische Charakterisierung der perizentrischen Inversion des Chromosoms 9- Bruchpunktanalyse und zusätzliche Strukturaberrationen. Dissertationzum Erwerb des Doktorgrades der Medizin an der Medizinischen Fakultät der Ludwig-Maximilians-Universität zu München. 2017.
163. Kingston HM, Ledbetter DH, Tomlin PI, Gaunt KL. Miller-Dieker syndrome resulting from rearrangement of a familial chromosome 17 inversion detected by fluorescence in situ hybridisation. J Med Genet (1996) 33:69-72.
164. Ilgin Ruhi H, Tükün A, Karabulut H, Bayazit P, Bökesoy I. A Down syndrome case with a karyotype of 46,XY,rec(21)dup(21q)inv(21)(p11q22) derived from paternal pericentric inversion of chromosome 21. Clin Genet (2001) 59:368-70.
165. Machuca STV, Ore IG, Loo MLF, Murgado JZ, Gonzales DT, Rosales LL, Aguilar LC. Partial trisomy 4p recombinant chromosome 4 caused by pericentric inversion of paternal origin. Rev Peru Matern Perinat (2015) 4:64-68.
166. Gazala J, Amithkumar IV, Sabina J, Praveena KK, Sujatha J. Prenatal diagnosis in pericentric inversion 6. Int J Hum Genet (2010) 10:175-8.
167. Schroer RJ, Culp DM, Stevenson RE, Potts WE, Taylor HA, Simensen RJ. Duplication-deletion syndrome in a family with pericentric inversion of chromosome 6. Clin Genet (1980) 18:83-7.
168. Magenis RE, Chamberlin J, Overton K, Lovrien E. Linkage relationships of HLA and a familial chromosome 6 inversion (pter leads to p23::q23 leads to qter): lack of dose effect in duplication-deficient offspring. Cytogenet Cell Genet (1978) 22:418-20.
169. Pearson G, Mann JD, Bensen J, Bull RW. Inversion duplication of chromosome 6 with trisomic codominant expression of HLA antigens. Am J Hum Genet (1979) 31:29-34.
170. Kamiya TY. Caracterização do espectro fenotípico de pacientes com fissuras labiopalatinas associadas a múltiplas anomalias congênitas e alterações cromossômicas estruturais. Universidade de Sao Paulo Hospital. (2009) PhD thesis
171. Patil SJ, Phadke SR. Pericentric inversion causing duplication and deletion of chromosome region 13q22 --> qter in the offspring. Am J Med Genet A (2007) 143A:82-4.
172. Maeda T, Ohno M, Takada M, Nishida M, Tsukioka K, Tomita H. Turner’s syndrome with a duplication deficiency X chromosome derived from a maternal pencentric inversion X chromosome. Clin Genet (1979) 15:259-66.
173. Dutrillaux B, Laurent C, Robert JM, Lejeune J. [Pericentric inversion, inv(10), in a mother and aneusomy by recombination, inv(10), rec(10), in her son (author's transl)].Cytogenet Cell Genet (1973) 12:245-53. French.
174. Gadzicki D, Baumer A, Wey E, Happel CM, Rudolph C, Tönnies H, Neitzel H, Steinemann D, Welte K, Klein C, Schlegelberger B. Jacobsen syndrome and Beckwith-Wiedemann syndrome caused by a parental pericentric inversion inv(11)(p15q24). Ann Hum Genet (2006) 70(Pt 6):958-64.
175. Demars J, Rossignol S, Netchine I, Lee KS, Shmela M, Faivre L, Weill J, Odent S, Azzi S, Callier P, Lucas J, Dubourg C, Andrieux J, Le Bouc Y, El-Osta A, Gicquel C. New insights into the pathogenesis of Beckwith-Wiedemann and Silver-Russell syndromes: contribution of small copy number variations to 11p15 imprinting defects. Hum Mutat (2011) 32:1171-82.
176. Lagier-Tourenne C, Ginglinger E, Alembik Y, De Saint Martin A, Peter MO, Dulucq P, Jonveaux P, Jeandidier E. Two cousins with partial trisomy 12q and monosomy 12p recombinants of a familial pericentric inversion of the chromosome 12. Am J Med Genet A (2004) 125A:77-85.
177. Hauksdóttir H, Halldórsson S, Jensson O, Mikkelsen M, McDermott A. Pericentric enversion of chromosome no. 13 in a large family leading to duplication deficiency causing congenital malformations in three individuals. J Med Genet (1972) 9:413-21.
178. Kaiser P, Förster W, Steuernagel P, Hillig U, Herberg KP. Familial pericentricinversion (14) (p11;q24) with a rec dup(q) in one offspring. Clin Genet (1984) 26:73-6.
179. O'Connor R, Al-Murrani A, Aftimos S, Asquith P, Mazzaschi R, Eyrolle-Guignot D, George AM, Love DR. Pure duplication of the distal long arm of chromosome 15 with ebstein anomaly and clavicular anomaly. Case Rep Genet (2011) 2011:898706.
180. Okubo Y, Siddle K, Firth H, O'Rahilly S, Wilson LC, Willatt L, Fukushima T, Takahashi S, Petry CJ, Saukkonen T, Stanhope R, Dunger DB. Cell proliferation activities on skin fibroblasts from a short child with absence of one copy of the type 1 insulin-like growth factor receptor (IGF1R) gene and a tall child with three copies of the IGF1R gene. J Clin Endocrinol Metab (2003) 88:5981-8.
181. Tatton-Brown K, Pilz DT, Orstavik KH, Patton M, Barber JC, Collinson MN, Maloney VK, Huang S, Crolla JA, Marks K, Ormerod E, Thompson P, Nawaz Z, Lese-Martin C, Tomkins S, Waits P, Rahman N, McEntagart M. 15q overgrowth syndrome: a newly recognized phenotype associated with overgrowth, learning difficulties, characteristic facial appearance, renal anomalies and increased dosage of distal chromosome 15q. Am J Med Genet A (2009) 149A:147-54.
182. Vaglio A, Milunsky A, Huang XL, Quadrelli A, Mechoso B, Quadrelli R. A fourteen years follow-up of a case of partial trisomy 12q and monosomy 12p recombinants of a familial pericentric inversion of chromosome 12: clinical, cytogenetic and molecular observations. Eur J Med Genet (2007) 50:224-32.
183. Stoler JM, Leach NT, Donahoe PK. Case 36-2004: A 23-day-old infant with hypospadias and failure to thrive. N Engl J Med (2004) 351:22.
184. Del Casal P-J, Artigas Lopez M, Moreno Laguna S, Bengoa Alonso A,. Yoldi Petri E, Ramos-Arroyo MA. Cerebellar hypoplasia: a new ¢nding indup(10p)/del (10q) syndrome. Chromosome Res (2005) 13 (Suppl 1):32 (1.37-P)
185. Lasan Trcic R, Letica L, Crkvenac Gornik K, Tonkovic I, Muzinic D, Begovic D. Partial trisomy 17q resulting from a familial pericentric inversion. Chromosome Res (2005) 13 (Suppl 1):38 (1.52-P)
186. Nowakowka B, Bocian E, Patel A, Stankiewicz P, Cheung SW, Cai WW. Validation and application of genome-wide array comparative genomic hybridization (array-CGH) with 21,500 BAC clones in cytogenetic analysis. Chromosome Res (2005) 13 (Suppl 1):213 (14.27-P)
187. Lee MJ, Park SH, Shim SH, Moon MJ, Cha DH. Prenatal diagnosis and molecular cytogenetic characterization of partial dup(18q)/del(18p) due to a paternal pericentric inversion 18 in a fetus with multiple anomalies. Taiwan J Obstet Gynecol (2019) 58:318-23.
188. Israëls T, Hoovers J, Turpijn HM, Wijburg FA, Hennekam RC. Partial deletion of 18p and partial duplication of 18q caused by a paternal pericentric inversion. Clin Genet (1996) 50:520-4.
189. Patil S, Hanson J, Zellwegner H. Duplication–deletion of chromosome 3 syndrome. Am J Hum Genet (1978) 30:A89.
190. Mulcahy MT, Pemberton PJ, Sprague P. Trisomy 3q: two clinically similar but cytogenetically different cases. Ann Genet (Paris) (1979) 22:217-20.
191. Polivkova Z, Kucerova M, Dolanska M, Hresova M, Kofer J. Podobnost syndromuz castesne trisomie dlouhych ramen 3. Chromosomua syndrom Cornelia de Lange. Cs Pediatr (1988) 43:723-6.
192. Warren RJ, Haijanpour MJ, Lewis M, Romine LS. Aneusomy by recombination of a pericentric inverted chromosome 4. Am J Hum Genet (1988) 43:A126.
193. Wiegand W. Augenveränderungen bei partieller Trisomie 4p. Fortschr Ophtalmol (1986) 83:317-9.
194. Kobori JA, Seto-Donlon S, Gregory T, Bangs D, Donlon TA. A case of monosomy 4p and trisomy 4q derived from a meiotic recombination. Am J Hum Genet (1993) 53:A1573.
195. Winsor EJ, Palmer CG, Ellis PM, Hunter JL, Ferguson-Smith MA. Meiotic analysis of a pericentric inversion, inv(7) (p22q32), in the father of a child with a duplication-deletion of chromosome 7. Cytogenet Cell Genet (1978) 20:169-84.
196. Stanley RG, Dev VG, Buttler MG, Phillips JA. Partial 8q trisomy and 8p monosomy resulting from inversion in paternal chromosomes8. Am J Hum Genet (1985) 37:A118.
197. Mattei JF, Mattei MG, Balestrazzi P, Giraud F. Familial pericentric inversion of chromosome 9, INV(9)(p22q32) with recurrent duplication-deletion. Clin Genet (1983) 24:220-2.
198. Mejia-Baltodano G, Bobadilla L, Gonzales RM, Barros-Nunez P. High recurrence of recombinations in a family with pericentric inversion of chromosome 18. Ann Genet (Paris) (1997) 40:164-8.
199. Rethore MO, Dutrillaux B, Job JC, Lejeune J. Trisomie 4p par aneusomie de recombinaison d'une inv (4) (p14q35). Ann Genet (Paris) (1974): 17:109-14.
200. Bocchini V, Armellini R, Mariucci G, Migliorini G. Pericentric inversion of chromosome 4 through three generations. Riv Biol (1983) 76:103-5.
201. Murray J, Patil S, Fatemi C, Aschbacher A, Buetow K, Ferell R, Miles J, Smith M, Carlock L. Effect of a large pericentric inversion of chromosome 4 on meiotic recombination. Am J Hum Genet (1985) 37:A109.
202. Dallapiccola B, Capra L, Preto G, Covic M, Dutrillaux B. Inversion pericentrique du 4: inv (4) (p13q35) et trisomie du bras court du 4 par aneusomie de recombinaison. Ann Genet (Paris) (1974) 17: 115-8.
203. Biesecker LG, Rosenberg M, Dziadzio L, Ledbetter DH, Ning Y, Sarneso C, Rosenbaum K. Detection of a subtle rearrangement of chromosome 22 using molecular techniques. Am J Med Genet (1995) 58:389-94.
204. Putoux A, Labalme A, André JM, Till M, Schluth-Bolard C, Berard J, Bertrand Y, Edery P, Putet G, Sanlaville D. Jacobsen and Beckwith-Wiedemann syndromes in a child with mosaicism for partial 11pter trisomy and partial 11qter monosomy. Am J Med Genet A (2013) 161A:331-7.
